# d-allulose protects against diabetic nephropathy progression in Otsuka Long-Evans Tokushima Fatty rats with type 2 diabetes

**DOI:** 10.1371/journal.pone.0263300

**Published:** 2022-01-31

**Authors:** Misato Niibo, Akane Kanasaki, Tetsuo Iida, Keisuke Ohnishi, Taro Ozaki, Kazuya Akimitsu, Tetsuo Minamino

**Affiliations:** 1 Research and Development, Matsutani Chemical Industry Co., Ltd, Itami City, Hyogo, Japan; 2 Department of Cardiorenal and Cerebrovascular Medicine, Faculty of Medicine, Kagawa University, Miki, Kagawa, Japan; 3 International Institute of Rare Sugar Research and Education & Faculty of Agriculture, Kagawa University, Miki, Kagawa, Japan; Max Delbruck Centrum fur Molekulare Medizin Berlin Buch, GERMANY

## Abstract

d-allulose is a rare sugar that has been reported to possess anti-hyperglycemic effects. In the present study, we hypothesized that d-allulose is effective in attenuating the progression of diabetic nephropathy in the Otsuka Long-Evans Tokushima Fatty (OLETF) rat model of type 2 diabetes mellitus. Drinking water with or without 3% d-allulose was administered to OLETF rats for 13 weeks. Long-Evans Tokushima Otsuka rats that received drinking water without d-allulose were used as non-diabetic control rats. d-allulose significantly attenuated the increase in blood glucose levels and progressive mesangial expansion in the glomerulus, which is regarded as a characteristic of diabetic nephropathy, in OLETF rats. d-allulose also attenuated the significant increases in renal IL-6 and tumor necrosis factor-α mRNA levels in OLETF rats, which is a proinflammatory parameter. Additionally, we showed that d-allulose suppresses mesangial matrix expansion, but its correlation with suppressing renal inflammation in OLETF rats should be investigated further. Collectively, our results support the hypothesis that d-allulose can prevent diabetic nephropathy in rats.

## Introduction

Diabetic nephropathy (DN) is a severe diabetic microvascular complication with high morbidity and mortality rates among patients with diabetes and is the leading cause of end-stage renal disease in developed countries, including Japan [1]. Chronic high glucose levels induce the production of advanced glycation end products and enhance the expression of growth factors, both of which lead to DN [2–4]. Studies have reported that glomerular cells from diabetic animals and mesangial cells cultured in high-glucose media exhibit high proliferation rates [5, 6]. Mesangial matrix expansion is a major morphological characteristic of DN [7].

d-allulose (d-*ribo*-2-hexylose, also known as d-psicose) is an epimer of d-fructose and is a rare, natural monosaccharide that is isomerized at the C-3 position of d-fructose. It has zero calories and approximately 70% sweetness relative to sucrose [8]. In humans, approximately 70% of ingested d-allulose is absorbed in the small intestine and excreted through urine without being metabolized further [9]. It was reported that d-allulose intake for 48 weeks did not aggravate renal function in individuals with high levels of low-density lipoprotein cholesterol and deteriorating glucose tolerance [10]. Studies have reported that d-allulose reduces postprandial blood glucose levels by suppressing α-glucosidase [11] and enhancing hepatic glucokinase translocation [12]. Additionally, d-allulose has been reported to show beneficial anti-diabetic effects in Otsuka Long-Evans Tokushima Fatty (OLETF) rats [12–14]. However, the effect of d-allulose on the progression of diabetic complications, including those related to kidney function, has not been determined. Because OLETF rats have been previously used as a model of DN [15], we sought to verify our hypothesis that d-allulose is effective in attenuating the progression of DN using this rat model.

## Materials and methods

### Materials

d-allulose was supplied by the International Institute of Rare Sugar Research and Education, Kagawa University (Miki, Kagawa, Japan). The purity of d-allulose exceeded 99%, according to the information provided by the supplier.

### Animal experiments

The animal experiments were performed according to Rules of Animal Experiment in Kagawa University, and approved by the Animal Care and Use Committee of Kagawa University (approval No: 2019–19627). All efforts were made to minimize suffering.

Six-week-old male OLETF rats and Long-Evans Tokushima Otsuka (LETO) rats were purchased from Hoshino Laboratory Animals, Inc. (Bando, Ibaraki, Japan). All rats were individually housed in separate cages under controlled conditions (temperature: 22 ± 2°C; humidity: 55% ± 5%) with 12 h light/dark cycles. Animals were provided with water and a commercial rodent diet (MF diet; Oriental Yeast Co., Ltd., Itabashi, Tokyo, Japan) *ad libitum* until the OLETF rats developed diabetes. The nutritional composition of the diet is presented in Table 1. We observed the animals daily to check for abnormal appearance, behavior, and excretion. As an indicator of animal health, the animals were weighed once a week and food intakes were measured once every 3 days. To evaluate the progression of diabetes, a previous report indicated that male OLETF rats become definitively diabetic at 25 weeks of age [16]. To verify this, we collected blood from the tips of the rat tails and measured the non-fasting blood glucose levels of the rats at 23 weeks of age and fasting blood glucose levels at 25 weeks of age using a glucometer (ARKRAY, Inc., Kyoto, Kyoto, Japan). Results showed that 23-week-old OLETF rats had significantly higher blood glucose levels (336 mg/dL) than those of the LETO rats (107 mg/dL). We therefore determined that all 27-week-old OLETF rats had already developed diabetes, and categorized OLETF rats into an OLETF control group (O-C, n = 10) and an OLETF-allulose group (O-A, n = 10) based on their body weight (initial body weight in Table 2). Male LETO rats (n = 6) were used as a negative control. Based on previous studies on the anti-diabetic effect of d-allulose [12–14] using OLETF rats, drinking water without d-allulose was administered to the O-C and LETO groups and drinking water containing 3% (w/v) d-allulose was administered to the O-A group for 13 weeks. The dose of d-allulose was set at 3% to correspond to a quantity close to an intake amount in humans based on doses in previous clinical trials [10, 17, 18] and maximum non-effect level in humans [19]. For the calculation of a human dose to an animal dose, an animal to human conversion formula based on body surface area [20] was used. The body weight and drinking quantity of OLETF in a previous study [14] was referred in the formula. Non-fasting and 16-h fasting blood glucose levels in the blood obtained from the tips of the tails at 4, 8, and 12 weeks after grouping were measured using a glucometer. All rats were maintained in individual metabolic cages to collect 24-hour urine samples at 12 weeks after grouping. After 16-hour fasting, all 40-week-old rats were sacrificed by blood extraction from the abdominal aorta under deep anesthesia with 5% isoflurane in accordance with a guideline of euthanasia by the American Veterinary Medical Association. The blood was centrifuged at 1,750 *g* for 15 min at 4°C using Laboratory centrifuge 5922 (KUBOTA Corp., Tokyo, Japan) to obtain plasma. The mice kidneys, liver, and perirenal white adipose tissue (WAT) were removed, weighed, and flash-frozen in liquid nitrogen. The samples were stored at −80°C until further analyses. The kidney parts were fixed in 10% neutral buffered formalin followed by paraffin embedding for histological examination.

**Table 1:** Nutritional composition of the diet.

Nutrient		Value per 100 g of diet
Energy	kcal	359
Carbohydrates	g	55.3
Fiber	g	2.8
Total lipid	g	5.1
Protein	g	23.1
Vitamin	g	0.650
Mineral	g	3.25

**Table 2:** Body weight, food and water intake, tissue weights, and plasma and urine parameters of the experimental rats.

	LETO			O-C			O-A			*P-*value
Initial body weight (g)	460	±	9^a^	567	±	16^b^	567	±	15^b^	<0.001
Final body weight (g)	463	±	9^a^	539	±	31^a^^b^	576	±	17^b^	0.017
Body weight gain (g)	2.85	±	3.73	-28.4	±	20.1	9.58	±	16.52	0.248
Food intake (g/day)	23.1	±	0.6^a^	36.3	±	1.3^b^	29.5	±	0.8^c^	<0.001
Water intake (mL/day)	29.6	±	1.0^a^	93.3	±	16.4^b^	46.2	±	8.5^a^	0.004
Tissue weights (g/100 g BW)										
Kidney	0.490	±	0.008^a^	0.694	±	0.055^b^	0.629	±	0.041^a^^b^	0.027
Liver	2.39	±	0.02^a^	3.66	±	0.15^b^	3.56	±	0.19^b^	<0.001
Perirenal WAT	2.15	±	0.07^a^	6.77	±	0.60^b^	7.55	±	0.26^b^	<0.001
Plasma parameters (mg/dL)										
Creatinine	0.450	±	0.019^a^	0.270	±	0.015^b^	0.252	±	0.008^b^	<0.001
BUN	15.7	±	0.7	18.6	±	1.9	17.1	±	2.4	0.661
Urine parameters										
NAG (U/24 h)	0.085	±	0.014^a^	0.486	±	0.052^b^	0.411	±	0.024^b^	<0.001
Albumin (mg/24 h)	0.137	±	0.032^a^	141	±	46^b^	66.5	±	11.8^a^^b^	0.025

The data show mean ± standard error (SE) (n = 10 for OLETF rats and n = 6 for LETO rats).

^abc^Different letters in the same column indicate statistical difference, *P* < 0.05, Tukey’s multiple comparison test. *P*-values were detected using one-way ANOVA.

BW, body weight; WAT, white adipose tissue; BUN, blood urea nitrogen; NAG, N-acetyl glucosaminidase; LETO, Long-Evans Tokushima Otsuka; OLETF, Otsuka Long-Evans Tokushima fatty; O-C, OLETF control; O-A, OLETF d-allulose.

### Biochemical assay

Plasma creatinine measurement was performed by the enzymatic method at Oriental Yeast Co., Ltd. The level of blood urea nitrogen (BUN) in the plasma was measured in duplicate using a commercial kit (Catalog No.: K024-H1) from Arbor assays (Ann Arbor, MI, USA). Plasma insulin levels and urinary albumin levels were measured in duplicate using enzyme-linked immunosorbent assay kits (Catalog No.: M1101, AKRAL-120) made by the Morinaga Institute of Biological Science, Inc. (Yokohama, Kanagawa, Japan) and by Shibayagi Co., Ltd. (Shibukawa, Gunma, Japan), respectively. Urinary N-acetylglucosaminidase (NAG) levels were measured using a commercial kit (Catalog No.: DNAG-10) from BioAssay Systems (Hayward, CA, USA).

### Histological examination

Histological examination was performed at the Biopathology Institute Co., Ltd. (Kunisaki, Oita, Japan). The paraffin-embedded kidney parts were used to prepare 3–4 μm thick sections, followed by mounting on a glass slide and staining with periodic acid-Schiff (PAS). The glomeruli stained with PAS were photographed with a microscope (magnification × 400). The PAS-positive area was measured using an image analysis software (WinROOF 2018; Mitani Corporation, Toshima, Tokyo, Japan). The renal tubules were excluded, and the total area of 50 glomeruli and the area of mesangial matrix were determined for each rat. Then, the average proportions of mesangial matrix area per glomerular area were calculated.

### Real-time Reverse Transcription Polymerase Chain Reaction (RT-PCR)

Total RNA was extracted from the renal cortical tissues and purified using RNAiso Plus (Catalog No.: 9108, Takara Bio, Kusatsu, Shiga, Japan). Reverse transcription was performed using a High-Capacity cDNA Reverse Transcription Kit (Catalog No.: 4368814, Thermo Fisher Scientific Inc., Waltham, MA, USA) under the following conditions: 10 min at 25°C, 120 min at 37°C, and 5 min at 85°C. Quantitative gene expression analysis was performed with Real-time PCR Systems (Life Technologies Japan Ltd., Minato, Tokyo, Japan) using a PowerUp^™^ SYBR^™^ Green Master Mix (Catalog No.: A25780, Thermo Fisher Scientific Inc. The sequence of each primer was as follows: Rat interleukin (IL)-6 (NM_012589.1) forward: 5′ TTGCCTTCTTGGGACTGATGT 3′ and reverse: 5′ TGTTGTGGGTGGTATCCTCTG 3′; rat tumor necrosis factor (TNF)-α (NM_012675.2) forward: 5′ GCTCCCTCTCATCAGTTCCA 3′ and reverse: 5′ CTCCTCTGCTTGGTGGTTTG 3′; rat glyceraldehyde 3-phosphate dehydrogenase (GAPDH) (NM_017008) forward: 5′ TGAACGGGAAGCTCACTGG 3′ and reverse: 5′ GCTTCACCACCTTCTTGATGTC 3′. For all RT-PCR analyses, *GAPDH* mRNA was used to normalize RNA levels. All data were expressed as relative differences between the LETO group and the other groups.

### Statistical analyses

All values are presented as the mean ± standard error (SE). Significant differences among the groups were determined by one-way or two-way repeated measures analysis of variance (ANOVA) and Tukey’s multiple comparison test as the post-hoc test, using the SPSS software (version 27, IBM analytics, USA). Results with a *P*-value < 0.05 were considered to be statistically significant.

## Results

### Effects of d-allulose on body weight, food intake, water consumption, tissue relative weight, and kidney parameters

Table 2 summarizes the body weights, food intake, water consumption, tissue relative weights, plasma parameters, and urinary parameters of the experimental animals. While there were no significant differences between the LETO and O-C groups with respect to final body weights, the rats in the O-A group indicated higher levels of final body weight than LETO group. Food intake and water consumption levels of the rats in the O-C group were significantly higher than those of the rats in the LETO and O-A groups. The relative kidney weights of the rats in the O-C group were significantly higher than those of the LETO rats. However, no significant differences were observed between the relative kidney weights of the O-A group and the LETO and O-C groups. The relative liver and perirenal WAT weights of the rats in the O-C and O-A groups were significantly higher than those of LETO rats. There were significant decreases in the levels of plasma creatinine in the OLETF rats compared with LETO rats, which is frequently observed with the over-filtration of creatinine at the glomerulus in the early stage of DN. The levels of NAG, which is a marker of damage in the renal tubule in the early stage of DN, were significantly increased in the OLETF group compared to the LETO group; and urine albumin excretion in the OLETF rats was found to be significantly higher than that in the LETO rats. However, this increase was suppressed by d-allulose administration.

### Non-fasting and fasting glucose and insulin levels

**Fig 1:**
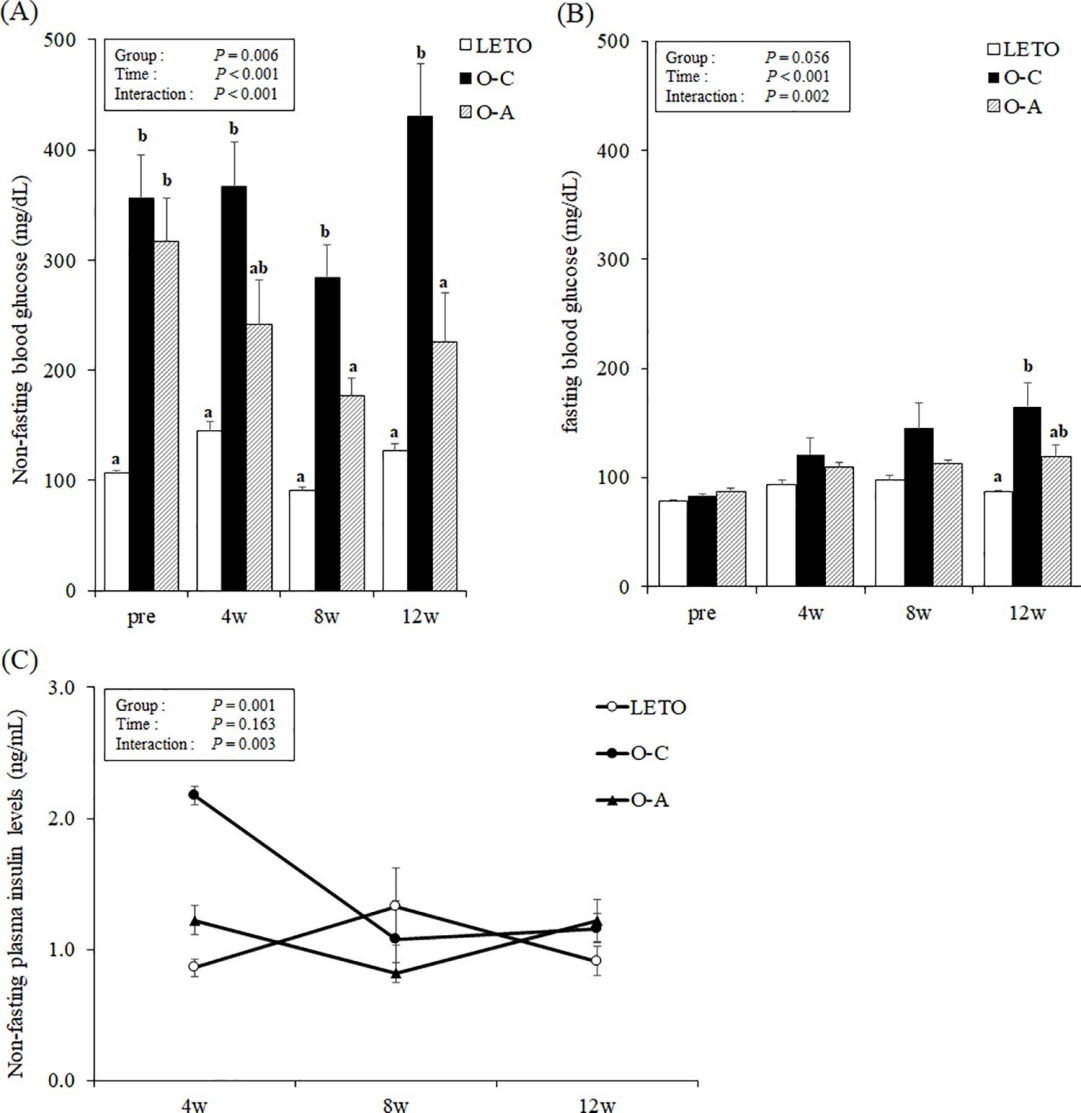
Periodic changes in the biochemical parameters relating glucose metabolism in OLETF and LETO rats. (A) Non-fasting blood glucose. (B) Fasting blood glucose. (C) Non-fasting insulin levels. The data are presented as mean ± standard error (SE) (n = 10 for OLETF rats and n = 6 for LETO rats). The overall *P* value from the two-way repeated measures ANOVA with repeated measures is shown in the graph. ^ab^Different letters in the same column indicate statistical difference, *P* < 0.05, Tukey’s multiple comparison test. LETO, Long-Evans Tokushima Otsuka; OLETF, Otsuka Long-Evans Tokushima fatty; O-C, OLETF control; O-A, OLETF d-allulose.

Time × treatment interactions in the non-fasting and fasting glucose and insulin levels were observed via two-way repeated measures ANOVA (Fig 1). At pre-treatment, the non-fasting blood glucose levels of OLETF rats were significantly higher than those of LETO rats. There was no significant difference between the non-fasting blood glucose levels of the O-C and O-A groups at pre-treatment, and between the O-A group and the LETO and O-C groups at 4 weeks. The non-fasting blood glucose levels in the LETO and O-A groups were significantly lower than those in the O-C group at 8 and 12 weeks after commencement of treatment, respectively (Fig 1A). There were no differences in the fasting blood glucose levels among the three groups at pre-treatment and at 4 and 8 weeks after the onset of treatment. At 12 weeks, the rats in the O-C group had higher fasting blood glucose levels than those in the LETO group, and the levels of blood glucose in the O-A group were not different from those in the LETO and O-C groups (Fig 1B). Despite the interaction of time × treatment in plasma insulin levels by two-way repeated measures ANOVA, no differences in insulin levels were observed among the three groups at all time points by Tukey post-hoc test (Fig 1C).

### Renal histological findings

**Fig 2:**
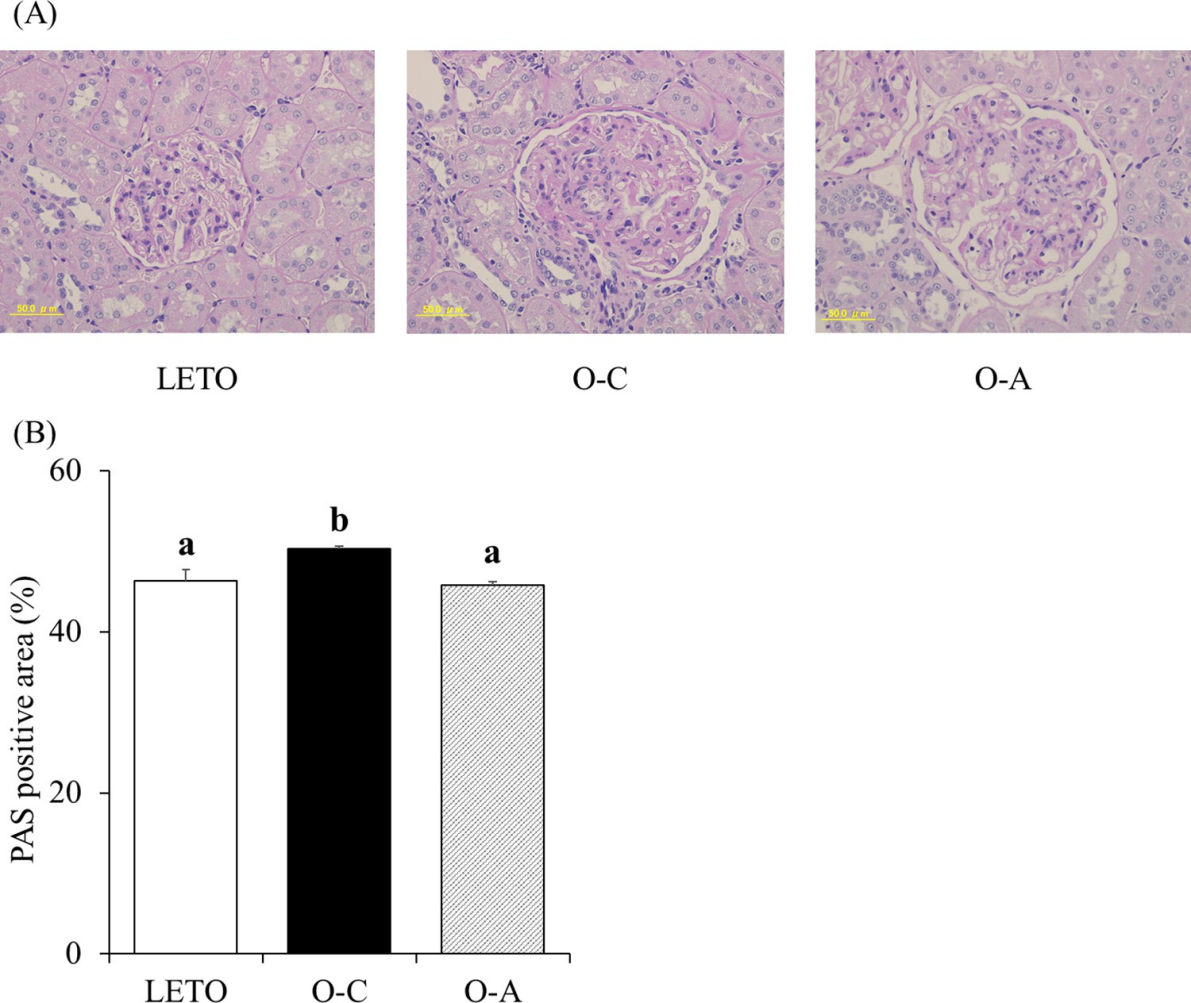
Histological analyses of kidneys in LETO and OLETF rats. (A) Kidney histological sections by the periodic acid-Schiff (PAS) stains (400 ×). (B) PAS-positive area within the total glomerular area. The data are presented as mean ± standard error (SE) (n = 10 for OLETF rats and n = 6 for LETO rats). ^ab^Different letters in the same column indicate statistical difference, *P* < 0.05, Tukey’s multiple comparison test. PAS, periodic acid-Schiff; LETO, Long-Evans Tokushima Otsuka; OLETF, Otsuka Long-Evans Tokushima fatty; O-C, OLETF control; O-A, OLETF d-allulose.

As shown in Fig 2, the PAS-positive area in the glomerulus, a marker of mesangial matrix expansion, differed significantly among the three groups by one-way ANOVA (*P* < 0.01). The PAS-positive area in the glomerulus significantly increased in the O-C group than in the LETO and O-A groups by Tukey’s multiple comparison. d-allulose treatment effectively suppressed mesangial expansion.

### mRNA levels of proinflammatory cytokines in the kidney

**Fig 3:**
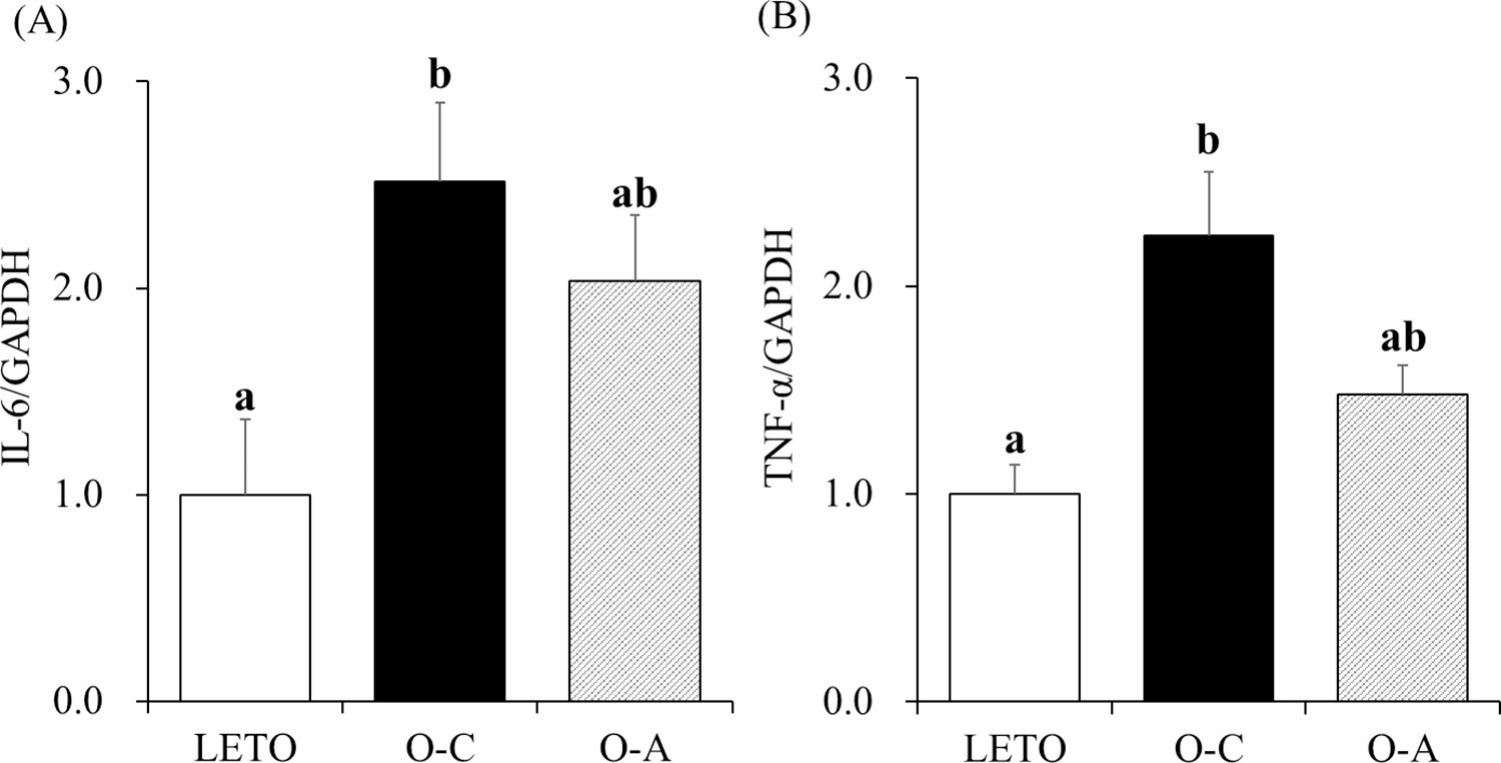
Effects of d-allulose on renal mRNA levels of proinflammatory cytokines. (A) mRNA levels of interleukin-6 (IL-6). (B) mRNA levels of tumor necrosis factor-α (TNF-α). The data are presented as mean ± standard error (SE) (n = 10 for OLETF rats and n = 6 for LETO rats). ^ab^Different letters in the same column indicate statistical difference, *P* < 0.05, Tukey’s multiple comparison test. GAPDH, Glyceraldehyde 3-phosphate dehydrogenase; LETO, Long-Evans Tokushima Otsuka; OLETF, Otsuka Long-Evans Tokushima fatty; O-C, OLETF control; O-A, OLETF d-allulose.

Fig 3 shows the mRNA levels of the proinflammatory cytokines IL-6 and TNF-α in the kidney. Significant differences in IL-6 and TNF-α mRNA levels were observed among three groups via one-way ANOVA. The results of one-way ANOVA followed by Tukey’s multiple comparison indicated that renal IL-6 and TNF-α mRNA levels in the rats of the O-C group significantly increased relative to the levels in the rats of LETO. The IL-6 and TNF-α mRNA levels in the rats of the O-A group did not differ from the levels in LETO rats.

## Discussion

The present study was conducted to confirm the anti-hyperglycemic and nephroprotective effects of d-allulose in diabetic OLETF rats. It was observed that d-allulose ameliorated the increase in blood glucose levels accompanied with progression of diabetes. Furthermore, DN progression was prevented by d-allulose consumption through the suppression of inflammation.

Although the levels of non-fasting blood glucose in the OLETF rats were higher than those in LETO rats, d-allulose significantly suppressed the increase in non-fasting blood glucose levels by the onset of diabetes. d-allulose also suppressed the significant increments in fasting blood glucose levels in O-C group rats relative to those in the LETO rats at 12 weeks. Previous studies on diabetic rat models have reported that d-allulose reduces blood glucose levels [12–14] by upregulating hepatic glucokinase translocation [12], improving insulin resistance, and protecting β-cells in the pancreas [13, 14]. Glucokinase has a lower affinity for glucose than other hexokinases and is known to metabolize glucose into glucose-6-phosphate in the postprandial state. As non-fasting blood glucose levels are generally higher than fasting blood glucose levels, it is expected that the attenuation of blood glucose levels due to d-allulose was more conspicuous in the postprandial state than in the fasting state, and these results are consistent with the finding of a previous study [14]. In this study, fasting and non-fasting blood glucose levels are used instead of hemoglobin A1c (HbA1c), which is generally used as an indicator of glycemic control, for evaluation of the glycemic control status because fasting and non-fasting blood glucose levels correlate with the levels of HbA1c in OLETF rats [14]. In a previous study, the acute administration of d-allulose resulted in lower blood glucose levels in normal mice that were in a fasting state after feeding on a high-fat diet [21]. On the other hand, in the present study, there was no significant decrease in blood glucose levels at 4 weeks after chronic treatment of d-allulose in OLETF rats that were in an ad libitum feeding state. The differences in the effects of d-allulose on blood glucose levels may be due to the administration method and/or dose of d-allulose, timing of glucose measurement, fasting status, presence or absence of diabetes, age, and genetic factors.

In the present study, no significant differences in insulin levels were observed through Tukey’s post-hoc test. However, the insulin levels displayed a difference among the groups (*P* = 0.0001) and interactions (*P* = 0.003) by analysis of the two-way repeated measures ANOVA due to the differences in overall trends. OLETF rats reportedly develop hyperglycemia and hyperinsulinemia in the early stage of diabetes because of insulin resistance, which eventually progresses to hypoinsulinemia as β-cells in islets get severely damaged [16, 22, 23]. Insulin secretion was thought to be reduced in the OLETF rats in the O-C group by the progression of diabetes in accordance with the results of a previous study (14). d-allulose appeared to influence a greater decrease in insulin levels at 4 to 8 weeks compared to the other groups for the amelioration of insulin resistance. d-allulose accordingly suppressed an increase in non-fasting blood glucose levels in the OLETF rats at 8 and 12 weeks despite no significant differences being observed in the non-fasting plasma insulin levels by Tukey’s post-hoc test. The attenuation of blood glucose levels in the O-A group was deduced to be the result of alleviated insulin resistance, in line with the findings of previous studies [12–14]. To elucidate the relationship between the blood glucose levels and the status of insulin resistance, additional research including indicators of insulin resistance, such as HOMA-IR, will be required.

In the present study, food intake was significantly reduced by d-allulose intake in OLETF rats. Because OLETF rats lack cholecystokinin-1 receptors, both their food intake rate and risk of developing metabolic disorders are high [24]. In addition, as a previous study has already shown that the restriction of feeding amount prevents the progression of diabetes in OLETF rats [25], it is reasonable to assume that d-allulose prevented a hyperglycemic state by suppressing overeating in the present study. Because the sweetness of d-allulose may potentially affect food intake, and consequently the other parameters of the O-A group, a sweetener with sweetness equivalent to that of d-allulose should be used as a comparison to d-allulose in future studies.

Although several previous studies have reported the anti-obesity effects of d-allulose, its administration did not affect the body weight or perirenal WAT weight of the OLETF rats in the present study. This may be because of the reduced scope of growth for the OLETF rats at 27 weeks of age, which is when we started the administration of d-allulose. Moreover, the rats in the present study were older than those used in previous studies [14]. The changes between the initial and final body weights of the OLETF rats were minimal in the present study. Another possibility is that the body weights of the rats in the O-C group did not increase but rather decreased because of diabetic progression, although there were no statistical differences in body weight gain between the O-C and O-A groups. Rats excrete glucose and protein into the urine according to the progression of diabetic status [26]. It is also well known that deterioration of diabetes results in the reduction of body weight. The administration of d-allulose is thought to ameliorate the progression of diabetes. The liver weights of OLETF rats increased compared with those in the LETO rats. It was suggested in a previous report that this increase in liver weight might be because a fatty liver is induced through the up-regulation of fatty acid synthesis in OLETF rats [27].

We found that the relative kidney weights of the O-C and O-A groups did not differ. However, we observed that the kidney weights of OLETF rats increased with the onset of diabetes, as determined by comparing the kidney weights of the LETO and O-C groups, although there was no significant increase in the kidney weights of the O-A group relative to the LETO group. Renal hypertrophy is a sign of diabetes in humans and experimental animals [28, 29]. In contrast to the present study, previous studies have reported that d-allulose often induced an increase in the kidney weight of normal rats without presenting any obvious toxicological findings through blood and histological analyses [30, 31]. However, this study showed that continuous consumption of d-allulose does not deteriorate renal hypertrophy induced by diabetes.

Our results clearly showed that d-allulose suppressed mesangial matrix expansion. Mesangial expansion, caused by the proliferation of mesangial cells, is a pathological feature of DN. Mesangial expansion leads to a reduced glomerular filtration rate and glomerulosclerosis [32, 33]. Therefore, it is indicated that suppression of proliferation in mesangial cells by d-allulose may prevent DN progression. The proliferation of mesangial cells is mainly caused by chronic hyperglycemia [6]. Because d-allulose exerted an anti-hyperglycemic effect in our study, we assumed that this effect contributed to the suppression of mesangial expansion. Another possibility is that d-allulose directly suppressed the expansion of the mesangial matrix through mechanisms such as the control of inflammation. Inflammation is known to be associated with mesangial cell proliferation [34]. Certain reports have shown that genes related to inflammation in adipose and hepatic tissues are downregulated by chronic administration of d-allulose [35, 36]. In the present study, we observed that renal TNF-α and IL-6 mRNA levels in the O-C rats were significantly higher than those in the LETO rats. The significant increases in IL-6 and TNF-α mRNA levels in the kidneys of OLETF rats relative to those in LETO rats were suppressed by d-allulose. Although there were no significant differences between the renal IL-6 and TNF-α mRNA levels of the O-C and O-A groups, the IL-6 and TNF-α mRNA levels in the O-A group were 19.2% and 34.0% lower than those in the O-C group, respectively. Previous studies have reported that the increases in serum IL-6 and TNF-α levels are significantly reduced in OLETF rats administered with d-allulose [14]. These results indicate that continuous consumption of d-allulose exerts anti-inflammatory effects not only on the plasma, adipose tissue, and liver, but also on the kidney, thereby preventing DN progression.

In this study, the albuminuria level in the O-C group was significantly higher than that in the LETO group; however, there was no significant difference between albuminuria levels of the O-A and LETO groups. Albuminuria is a known clinical feature of DN. However, albuminuria was not correlated with glomerular structural changes in a rat model of DN [37], indicating that albuminuria might not always be caused by mesangial matrix expansion in the early phase of DN. In addition, d-allulose did not affect the plasma creatinine and BUN levels in OLETF rats. Therefore, we concluded that d-allulose exerts its effects partly by suppressing the deterioration of a certain disease stage that eventually progresses to renal failure.

There are certain limitations to this study. d-allulose was administered orally via drinking water in this experiment, and so we could not determine an exact dose-effect relationship. According to the calculations of the administered amounts of d-allulose in this study, an intake amount in adult humans corresponds to approximately 23 g/60 kg body weight/day. To consider a correlation with the dose intended for human usage, an experiment using far fewer doses will be required. The exact mechanism by which mesangial expansion is reduced and the relationship between albuminuria levels and d-allulose could not be clearly elucidated. In addition, suitable animal models are required for further investigation. Further studies are needed to verify the nutritional or pharmaceutical uses of d-allulose in ameliorating diabetes and its complications and the elucidation of suppression mechanisms of mesangial matrix expansion by investigating related inflammation markers and macrophage infiltration in appropriate animal models using various d-allulose doses. We believe that our data will be useful in such future studies.

## Conclusion

The present study showed that d-allulose attenuates the increase in blood glucose levels associated with the attenuation of mesangial matrix expansion in a diabetes mellitus model of rats. Further research is required to investigate whether d-allulose could be useful to prevent the progression of DN in humans.

## Supporting information

S1 Table (XLSX)
